# Growth Rate, Dry Matter Accumulation, and Partitioning in Soybean (*Glycine max* L.) in Response to Defoliation under High-Rainfall Conditions

**DOI:** 10.3390/plants10081497

**Published:** 2021-07-21

**Authors:** Muhammad Ali Raza, Hina Gul, Feng Yang, Mukhtar Ahmed, Wenyu Yang

**Affiliations:** 1 College of Agronomy, Sichuan Agricultural University, Chengdu 611130, China; razaali0784@yahoo.com (M.A.R.); f.yang@sicau.edu.cn (F.Y.); 2 Sichuan Engineering Research Center for Crop Strip Intercropping System, Chengdu 611130, China; 3 Key Laboratory of Crop Ecophysiology and Farming System in Southwest China, Chengdu 611130, China; 4 National Research Center of Intercropping, The Islamia University of Bahawalpur, Bahawalpur 63100, Pakistan; 5 University Institute of Biochemistry and Biotechnology, PMAS Arid Agriculture University, Rawalpindi 46300, Pakistan; gulsatti@gmail.com; 6 Department of Agronomy, PMAS Arid Agriculture University, Rawalpindi 46300, Pakistan

**Keywords:** defoliation, leaf area, photoassimilate, crop management

## Abstract

The frequency of heavy rains is increasing with climate change in regions that already have high annual rainfall (i.e., Sichuan, China). Crop response under such high-rainfall conditions is to increase dry matter investment in vegetative parts rather than reproductive parts. In the case of soybean, leaf redundancy prevails, which reduces the light transmittance and seed yield. However, moderate defoliation of soybean canopy could reduce leaf redundancy and improve soybean yield, especially under high-rainfall conditions. Therefore, the effects of three defoliation treatments (T_1_, 15%; T_2_, 30%; and T_3_, 45% defoliation from the top of the soybean canopy; defoliation treatments were applied at the pod initiation stage of soybean) on the growth and yield parameters of soybean were evaluated through field experiments in the summer of 2017, 2018, and 2019. All results were compared with nondefoliated soybean plants (CK) under high-rainfall conditions. Compared with CK, treatment T_1_ significantly (*p* < 0. 05) improved the light transmittance and photosynthetic rate of soybean. Consequently, the leaf greenness was enhanced by 22%, which delayed the leaf senescence by 13% at physiological maturity. Besides, compared to CK, soybean plants achieved the highest values of crop growth rate in T_1_, which increased the total dry matter accumulation (by 6%) and its translocation to vegetative parts (by 4%) and reproductive parts (by 8%) at physiological maturity. This improved soybean growth and dry matter partitioning to reproductive parts in T_1_ enhanced the pod number (by 23%, from 823.8 m^−2^ in CK to 1012.7 m^−2^ in T_1_) and seed number (by 11%, from 1181.4 m^−2^ in CK to 1311.7 m^−2^ in T_1_), whereas the heavy defoliation treatments considerably decreased all measured growth and yield parameters. On average, treatment T_1_ increased soybean seed yield by 9% (from 2120.2 kg ha^−1^ in CK to 2318.2 kg ha^−1^ in T_1_), while T_2_ and T_3_ decreased soybean seed yield by 19% and 33%, respectively, compared to CK. Overall, these findings indicate that the optimum defoliation, i.e., T_1_ (15% defoliation), can decrease leaf redundancy and increase seed yield by reducing the adverse effects of mutual shading and increasing the dry matter translocation to reproductive parts than vegetative parts in soybean, especially under high-rainfall conditions. Future studies are needed to understand the internal signaling and the molecular mechanism controlling and regulating dry matter production and partitioning in soybean, especially from the pod initiation stage to the physiological maturity stage.

## 1. Introduction

The increasing human population is projected to raise food demand globally by 50% in 2030 [1]. The first four decades of the green revolution (from 1960 to 2000) witnessed substantial improvements in grain yields of staple food crops; however, the rate of improvement in crop yields has significantly declined in the past twenty years [2,3]. This decline was ascribed to the fact that the genetic approaches used for the green revolution are attaining their potential limits [4]. Besides, most of the remaining agricultural land utilized for agriculture production is easily erodible soils or environmentally sensitive regions, such as tropical forest areas [5]. Additionally, failure to enhance the crop yields on the currently available agricultural land will increase crop prices and the destruction of tropical forest areas for crop production [6]. At the same time, there is a continuing loss of agricultural land worldwide where urbanization occurs rapidly [7,8]. Therefore, modern and sustainable agronomic approaches are required now to fulfill the future demands for food crops [9], which we will face in the midcentury [10]. Thus, meeting the predicted world demand for food crops will require new crop production practices or methods beyond that employed in the green revolution [11,12].

Soybean (*Glycine max* L. Merr) is the major spring and summer food crop in the southwest of China [13,14]. Still, seed yield production varies mainly due to biotic, e.g., diseases [15], and abiotic stresses, e.g., nutrients [16], sunlight [17], and water [18]. The most critical growth phases for soybean to obtain better crop yield are from pod initiation to seed formation [19]. Previous studies have revealed that soybean would grow excessively under favorable growing conditions, especially its leaves [20]. Besides, there are heavy rains in the southwest of China during the monsoon season, which substantially increases the leaf area of soybean plants and decreases the photosynthetically active radiation transmittance in soybean canopy [21]. In line with this, researchers have confirmed leaf redundancy for soybean [5], and the top canopy leaves give shading to the more competent leaves in the middle and lower ranks for soybean plants [22]. Furthermore, shading from upper canopy leaves favors the early senescence of middle and bottom leaves [23,24], reducing the translocation of carbohydrates and nutrients to reproductive parts in soybean plants [25]. Moreover, these types of mutual shading conditions, especially during the reproductive phase of soybean, decrease the current photosynthetic rate and the availability of photoassimilates for developing pods and seeds, which ultimately decrease the final seed yield of soybean plants [24]. Thus, we hypothesized that extra leaf growth of soybean plants negatively affects the seed yield of soybean. It is crucial to determine the optimum leaf area of soybean to maximize crop yields, especially under high-rainfall conditions.

In past studies, researchers have reported the soybean response mechanisms to insect damage [26], weather or herbivory damage [27,28], and artificial defoliation [29]. These responses include reductions in light interception [26], photosynthetic characteristics [30], pod and seed number [31], seed size and weight [32], effective seed filling period [33], and seed yield [28,32]. However, insufficient information is available on how defoliation influences the photosynthetically active radiation (PAR) transmittance, dry matter accumulation, and partitioning in vegetative and reproductive parts, which ultimately affect the final pod number, seed number, and seed yield of soybean plants in field conditions. Determining the optimum leaf area for soybean, especially in high-rainfall conditions, is essential to obtain a better soybean yield. This will also help crop breeders and agronomists develop new soybean varieties and production practices to fulfill the projected food demands. Therefore, in the present study, we hypothesized that soybean produces extra leaves in the high-rainfall conditions, i.e., southwest of China, and a slight defoliation from soybean canopy would (a) improve the PAR transmittance at the soybean canopy, (b) delay the leaf senescence of remaining leaves by improving the light environment at the soybean canopy, and (c) increase the translocation of photoassimilate to pods and seeds, as well as the final seed yield of soybean under high-rainfall conditions. We evaluated these hypothesizes by comparing the defoliation of 15%, 30%, or 45% of the top leaves from the soybean canopy at the pod initiation stage with no defoliation treatment.

## 2. Results

### 2.1. Leaf Greenness and Leaf Area Index

Leaf greenness showed a significant variation from R_4_ to R_7_, while nonsignificant differences were found at R_3_ in all treatments (Table 1). At R_3_, R_4_, and R_5_, the average highest leaf greenness was measured in CK, while at R_6_ and R_7_, the highest leaf greenness was noted under treatment T_1_, whereas, at all sampling stages, the average lowest leaf greenness was noticed under the T_3_ treatment. Overall, compared to CK, treatment T_1_ increased the leaf greenness by 11% at R_6_ and by 22% at R_7_, suggesting that the reducing leaf area at the start of the reproductive phase (from R_3_ to R_5_) significantly enhanced the leaf greenness at the late reproductive stage (from R_6_ to R_7_), which delayed the leaf senescence in soybean plants under treatment T_1_.

**Figure 1:**
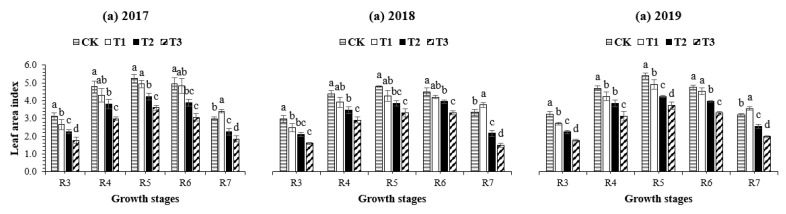
Leaf area index of soybean at the pod initiation stage (R_3_), full pod stage (R_4_), seed initiation stage (R_5_), full seed stage (R_6_), and physiological maturity stage (R_7_) as affected by different defoliation treatments during the summer season of 2017, 2018, and 2019. Treatment codes represent 100% leaf area (CK: control), 85% leaf area (T_1_), 70% leaf area (T_2_), and 55% leaf area (T_3_) from the soybean canopy. Means are averages over three replicates ± the standard error of the mean. Means that do not share the same letters in a column differ significantly at *p* < 0.05 using least significant differences (LSDs), calculated separately for each year.

The different defoliation treatments (T_1_, T_2_, and T_3_) significantly reduced the values of the leaf area index for the control treatment (CK), with the most significant (*p* < 0.05) reduction noticed under T_3_ (Figure 1). On average, at R_3_, R_4_, and R_5_, the maximum leaf area index was obtained in CK, while at R_6_ and R_7_, the maximum leaf area index was noted under treatment T_1_. In contrast, the mean minimum leaf area index was measured under treatment T_3_. Interestingly, relative to CK, treatment T_1_ significantly increased (by 14% in 2017, 13% in 2018, and 11% in 2019) the leaf area index of soybean plants at R_7_, indicating that the leaf senescence in soybean is directly associated with leaf greenness.

### 2.2. PAR Transmittance and Photosynthesis

As presented in Table 2, all defoliation treatments significantly improved the PAR transmittance of the soybean canopy at R_5_. The mean values for PAR transmittance revealed that the maximum PAR transmittance was noticed in T_3_, followed by T_2_, T_1_, and CK. Averaged across the years, compared to CK, the PAR transmittance was increased by 42% in T_1_, 98% in T_2_, and 146% in T_3_, indicating that soybean plants severely suffer from mutual shading of leaves.

All treatments significantly influenced the photosynthetic parameters of soybean plants (Table 2). The values of the photosynthetic rate (*Pn*), transpiration rate (*Tr*), and stomatal conductance (*Gs*) were significantly higher in T_1_, T_2_, and T_3_ than CK. However, defoliation treatments significantly reduced the intercellular CO_2_ concentration (*Ci*) values in soybean leaves compared to the corresponding values in the control treatment. At R_5_, treatment T_3_ increased *Pn*, *Tr*, and *Gs* by 62%, 88%, and 77%, respectively, compared CK in all years of study. In contrast, the average highest and lowest *Ci* values were noted in the CK and T_3_ treatments, respectively.

### 2.3. Dry Matter and Crop Growth Rate

**Figure 2:**
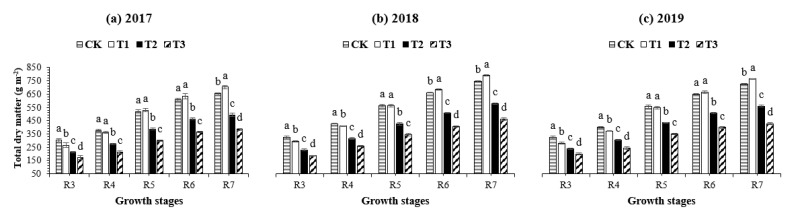
Total dry matter accumulation of soybean at the pod initiation stage (R3), full pod stage (R4), seed initiation stage (R5), full seed stage (R6), and physiological maturity stage (R7) as affected by different defoliation treatments during the summer season of 2017, 2018, and 2019. Treatment codes represent 100% leaf area (CK: control), 85% leaf area (T_1_), 70% leaf area (T_2_), and 55% leaf area (T_3_) from the soybean canopy. Means are averages over three replicates ± the standard error of the mean. Means that do not share the same letters in a column differ significantly at *p* < 0.05 using least significant differences (LSDs), calculated separately for each year.

The total dry matter accumulation (TDM) in soybean demonstrated an “S-type” curve in all defoliation treatments and the control treatment at different sampling stages (Figure 2). The TDM increased slowly from R_3_ to R_4_, demonstrated a steep increase from R_5_ to R_6_, and reached the highest level at R_7_. Averaged across the three years, at R_3_, R_4_, and R_5_, the maximum TDM was recorded under CK, while at R_6_ and R_7_, the maximum TDM was produced in the T_1_ treatment. In contrast, the minimum TDM was obtained in the T_3_ treatment. Furthermore, all treatments changed the TDM and influenced the partitioning of dry matter in vegetative and reproductive parts (Table 3). For example, compared to CK, treatment T_1_ significantly increased dry matter partitioning to vegetative and reproductive parts by 4% and 8% at R_7_, respectively, indicating that the higher green leaf at R_7_ (Table 2) maintained the high rate of photoassimilate translocation to reproductive parts. However, heavy defoliation (T_2_ and T_3_) significantly reduced the dry matter partitioning to vegetative parts (by 18% and 34%) and reproductive parts (by 27% and 45%) compared to the control treatment (CK).

In this experiment, the values of the crop growth rate among R_3_–R_4_, R–R_5_, R_5_–R_6_, and R_6_–R_7_ exhibited a similar trend (Table 4). In general, the crop growth rate of soybean was increased at the early reproductive growth stages and achieved its maximum value between the R_4_–R_5_ and R_5_–R_6_ stages; after that, it gradually reduced owing to leaf senescence in all treatments. On average, at all sampling stages, the highest crop growth rate was noticed in T_1_, while the lowest crop growth rate was recorded in treatment T_3_. Overall, between R_6_ and R_7_, T_1_ increased the crop growth rate of soybean plants by 52% in 2017, 24% in 2018, and 34% in 2019 compared to the corresponding values under CK.

### 2.4. Yield and Yield Components

Table 5 shows the yield (seed yield, kg ha^−1^) and yield components (number of pods m^−2^, number of seeds m^−2^, and 100-seed weight) of soybean under different treatments. Among the treatments, T_1_ produced the mean maximum soybean seed yield (2318.2 kg ha^−1^), whereas T_3_ had the mean minimum soybean seed yield (1428.1 kg ha^−1^) in all years of the experiment. Interestingly, a reduction of 15% of the leaf area at R_3_ increased the seed yield of soybean by 9% in T_1_ compared to nondefoliated soybean plants in CK. In this study, nonsignificant differences were noted among different treatments for the individual seed weight of soybean. However, different defoliation and control treatments showed significant effects on the pods m^−2^ and seeds m^−2^, with the highest values of 1012.7 pods m^−2^ and 1311.7 seeds m^−2^ being obtained in T_1_, followed by CK (823.8 pods m^−2^ and 1181.4 seeds m^−2^), T_2_ (680.9 pods m^−2^ and 953.3 seeds m^−2^), and T_3_ (527.6 pods m^−2^ and 789.7 seeds m^−2^). The dynamics of the number of pods m^−2^ and the number of seeds m^−2^ in 2019 under different treatments were consistent with those in the previous years. Overall, compared to CK, treatment T_1_ increased the number of pods m^−2^ and the number of seeds m^−2^ by 23% and 11%, respectively. Thus, treatment T_1_ significantly improved the final pod number and seed number in soybean plants, resulting in an improved seed yield of soybean.

### 2.5. Correlation Analysis

**Figure 3:**
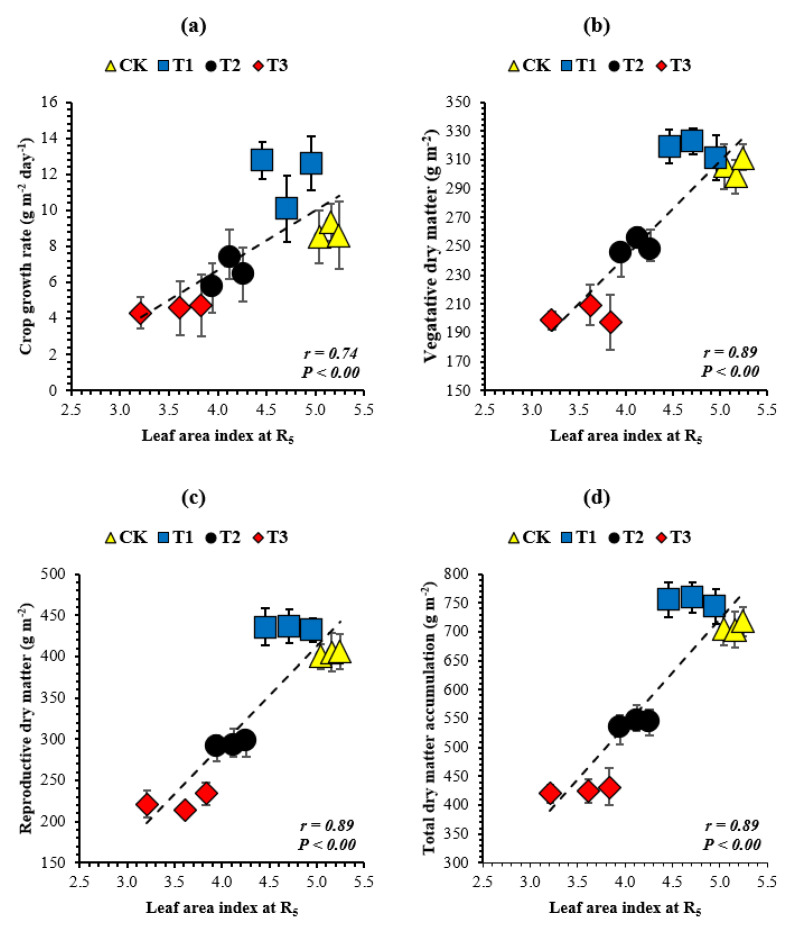
Relationship of the leaf area index at the R_5_ stage with the crop growth rate (**a**) and vegetative dry matter (**b**), reproductive dry matter (**c**), and total dry matter accumulation (**d**) of soybean at the R_7_ stage. Means are averages over three replicates ± the standard error of the mean. The dashed black line shows the relationship.

**Figure 4:**
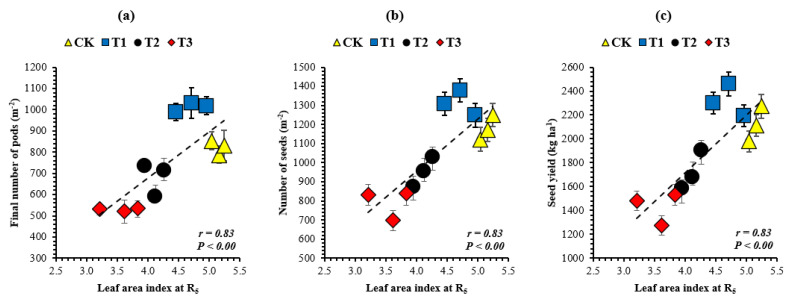
Relationship of the leaf area index at the R_5_ stage with the final number of pods (**a**), number of seeds (**b**), and seed yield (**c**) of soybean. Means are averages over three replicates ± the standard error of the mean. The dashed black line shows the relationship.

**Figure 5:**
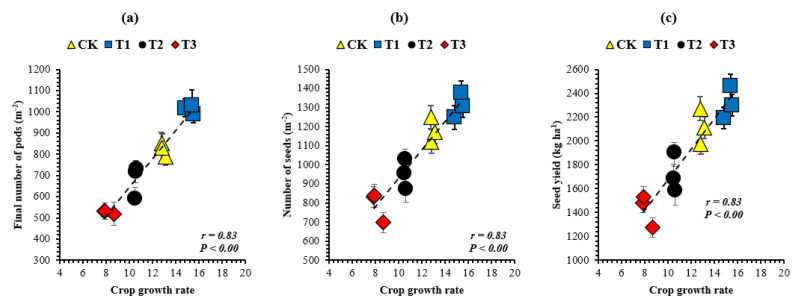
Relationship of crop growth rate during R_3_ to R_6_ (the critical period for seed setting) with the final number of pods (**a**), number of seeds (**b**), and seed yield (**c**) of soybean. Means are averages over three replicates ± the standard error of the mean. The dashed black line shows the relationship.

To recognize the indices wherein soybean growth and yield components were sensitive to the leaf area index, the relationship between decreasing leaf area index at the R_5_ stage and soybean growth (Figure 3) and yield components were investigated (Figure 4). Among the growth and yield components of soybean, the crop growth rate (g m^−2^ day^−1^), vegetative dry matter (g m^−2^), reproductive dry matter (g m^−2^), total dry matter accumulation (g m^−2^), final number of pods (m^−2^), number of seeds (m^−2^), and seed yield (kg ha^−1^) increased with increasing leaf area index. However, the highest values of all these parameters in all years of this study were noticed in treatment T_1_, where soybean plants produced 6% in 2017, 13% in 2018, and 10% in 2019, with less leaf area index than the corresponding soybean plants in the control treatment. We found that the crop growth rate (Figure 3a), vegetative dry matter (Figure 3b), reproductive dry matter (Figure 3c), total dry matter accumulation (Figure 3d), final number of pods (Figure 4a), number of seeds (Figure 4b), and seed yield (Figure 4c) were positively (*p* < 0.05) related to the leaf area index. Furthermore, the relationship between the crop growth rate during R_3_ to R_6_ (the critical period for seed setting) and soybean yield components was also investigated; and the final number of pods (m^−2^), number of seeds (m^−2^), and seed yield (kg ha^−1^) increased with increasing crop growth rate. However, the maximum yield components were noticed in treatment T_1_, where soybean plants achieved an 18% (mean of all the study years) higher crop growth rate than the corresponding soybean plants in the control treatment. We found that the final number of pods (Figure 5a), number of seeds (Figure 5b), and seed yield (Figure 5c) were positively (*p* < 0.05) related to the crop growth rate. The correlation coefficient among all the measured indices for the mean datasets was higher than 0.74 (*p* < 0.05).

## 3. Discussion

Crop leaves become more critical to growth and yield only when they act as sources, not as a sink, especially during the reproductive phase of crops [9]. Thus, crop yield is not always strongly correlated with leaf area, while crop leaves become a sink and are negatively correlated with seed yield [34]. Leaf senescence, leaf redundancy, and the low PAR transmittance at crop canopies are the primary reasons for converting crop leaves from source organs to sink organs. Leaf senescence is a natural process, which occurs during the lifecycle of crops. However, the early senescence of leaves significantly reduces crop yields [35,36]. Besides, leaf redundancy is defined as a relative increase in the number and size of leaves due to improper management practices (e.g., an improper (large) maturity group) or environmental factors (e.g., high rainfall). It changes the photoassimilate partitioning pattern from reproductive parts to vegetative parts and decreases crop yields [37]. Moreover, the low PAR transmittance in the middle and lower leaves is primarily due to the large canopy [34], high planting density [38], and plant height [39], which all together prevent light penetration at crop canopies, thereby causing a significant reduction in the current photosynthetic rate [9]. Therefore, the lower leaves cannot fulfill the plant demand for carbohydrates and nutrients, and they permanently act as a sink instead of a source [40]. However, the results of the present study revealed that the slight defoliation (T_1_) from the top of the soybean canopy significantly increased the PAR transmittance and photosynthesis of soybean compared to nondefoliated soybean plants. These positive responses also enhanced the leaf greenness of the remaining soybean leaves [41], which delayed the leaf senescence of soybean leaves by increasing their leaf greenness at R_6_ and R_7_. Consequently, the remaining lower leaves contributed carbohydrates and nutrients for a longer period to developing pods and seeds and remained a source throughout the reproductive phase. Whereas the heavy defoliation considerably increased the PAR transmittance and photosynthetic rate of soybean plants, this increment in the PAR transmittance and the photosynthetic rate did not compensate for the reduced total leaf area of soybean plants at all measuring stages in T_2_ and T_3_, indicating the decreased recovery growth from R_4_ to R_7_. Taken together, these results suggest that the slight defoliation at the start of the reproductive phase of soybean: (i) effectively reduced the leaf redundancy by reducing the photoassimilate consumption in the extra leaf growth under the high-rainfall conditions; and (ii) improved PAR transmittance at the soybean canopy, which delayed the leaf senescence caused by the mutual shading of leaves.

The leaf area of soybean is a critical index for obtaining a higher crop yield, and it is significantly influenced by abiotic (solar radiation and heavy rainfall) factors [42]. In addition, researchers had obtained the maximum soybean seed yield when their crops achieved a leaf area index between 3.5 and 4.0 at the beginning of the flowering stage under subtropical environments [31]. However, little is known about the optimum range of the leaf area index for soybean under low-light and high-rainfall conditions. Therefore, the determination of the optimum leaf area index, especially under high-rainfall conditions, is a first step to decrease the yield gaps in soybean production [42]. The experimental results demonstrated that the soybean plants appear to produce more leaves than essential for better crop yield under the high-rainfall condition. While new developing leaves from the R_4_ to R_7_ stages of soybean are detrimental for pod initiation and seed formation [43], extra crop foliage hinders the light penetration through the crop canopies [12]. Therefore, the benefit of having fewer leaves at the start of the reproductive phase is associated with higher PAR transmittance (Raza et al., 2019) and light use efficiency [44]. Similarly, in the previous study, the researchers confirmed that the increasing light intensity changed dry matter accumulation pattern in soybean by allocating more dry matter for pod initiation and seed formation [45], which significantly increased the pod and seed number in defoliated soybean plants compared to nondefoliated soybean plants [43]. Consequently, the amount of dry matter from the R_3_ to R_5_ stages is a critical factor determining yield and yield components in soybean [46]. These results indicate the potential to improve the soybean yield while increasing sustainability for light use efficiency, especially under high-rainfall conditions. Thus, it is possible that with increased photosynthesis and light use efficiency, soybean plants with a little lower leaf area at R_3_, R_4_, and R_5_ could save dry matter investment on the development and maintenance of extra vegetative parts. These dry matter savings could then be shifted to increase the final seed yield of soybean by increasing the pod initiation [5] and decreasing seed abortion [11].

At maturity, the pod and seed number of soybeans is the outcome of the balance between dry matter accumulation in vegetative and reproductive parts. In this study, a slight reduction in the leaf area of soybean plants at R_3_ significantly increased the number of pods and seeds through increased pod initiation and decreased seed abortion, respectively, by maintaining enough supply of carbohydrates to reproductive parts. Thus, under high-rainfall conditions, soybean requires a higher supply of photoassimilates to reduce pod abscission and seed abortion because, with an adequate supply of assimilates, each initiated pod and seed can develop into a mature pod and seed at final harvest [11]. However, mutual shading of leaves significantly reduces the net photosynthetic rate and carbohydrate supply to developing pods in soybean, especially at the pod initiation and seed initiation stages [43]. The slight defoliation in T_1_ improved the photosynthetic rate and maintained a higher supply of photoassimilates to reproductive parts during the reproductive phase of the soybean. Similarly, some studies on the predictive models incorporate the temporal profile of pod and seed initiations in the assimilate-based models [47,48]. Therefore, the present higher pod and seed number of soybeans in T_1_ than CK could be explained by assimilate-based models. Moreover, the results of this experiment exhibited that the better seed yield of soybean was measured in T_1_, followed by the CK, T_2_, and T_3_ treatments. Importantly, the leaf area index reduction in treatment T_1_ at R_3_ was 15%. It could only reduce the leaf area index of soybean plants by 9% and 5% (average of three years) at R_5_ and R_6_, respectively. Interestingly, it increased the leaf area index of soybean by 13% at R_7_ due to the delayed leaf senescence, resulting in a 9% increase in seed yield of soybean as compared to the control treatment. Therefore, we can conclude that the improved seed yield of soybean in T_1_ might be associated with the improved PAR transmittance and dry matter accumulation, leading to a higher partitioning of dry matter and nutrients to developing pods and seeds from R_3_ to R_7_. Delayed leaf senescence maintained the continuous assimilate supply, which reduced the pod abscission and seed abortion rate in soybean plants [11,43]. Therefore, the slight defoliation significantly increased the final pod and seed number, which increased the final seed yield. Moreover, the medium- or late-maturing soybean varieties tend to uptake more nutrients (nitrogen) from the soil under high-rainfall conditions, increasing the dry matter investment in vegetative parts, especially during the reproductive growth phase, as we observed in this study. Therefore, based on our results, we recommend 10–15% of defoliation from the top of the soybean canopy at the pod initiation stage, especially for medium- or late-maturing varieties, for higher PAR transmittance, dry matter partitioning towards reproductive parts, and seed yield of soybean plants. For this purpose, (i) leaf clipping machines can be developed to optimize soybean canopies for better crop yields, which will also reduce the leaf redundancy in soybean plants, especially under high-rainfall conditions; (ii) crop management practices (i.e., optimizing plant distribution through modifying plant population and row spacing) could be developed that could reduce the leaf redundancy in soybean plants; and (iii) some genetic modification of the leaf angle might be a plausible option for increasing light transmittance through the canopy, which will improve the current photosynthesis of soybean leaves and, finally, the seed yield. Furthermore, we can better control crop yields by regulating the crop canopies in field conditions [9], for instance: chemicals or plant growth regulators can be used at the appropriate time to control the vegetative growth (i.e., dry matter investment in new leaves during the reproductive phase) of soybean plants. Additionally, our optimal leaf removal findings can be applied generally to solve the problem of excessive vegetative growth of soybean, not only in heavy-rainfall regions but also in the regions where the active accumulated temperature is not enough, due to the sudden decrease of temperature in the late growing season (i.e., in Sichuan, the temperature drops sharply in September) and improper management (i.e., nitrogen and variety use), which do not allow promising results from short-duration varieties.

## 4. Materials and Methods

### 4.1. Experimental Site

**Figure 6:**
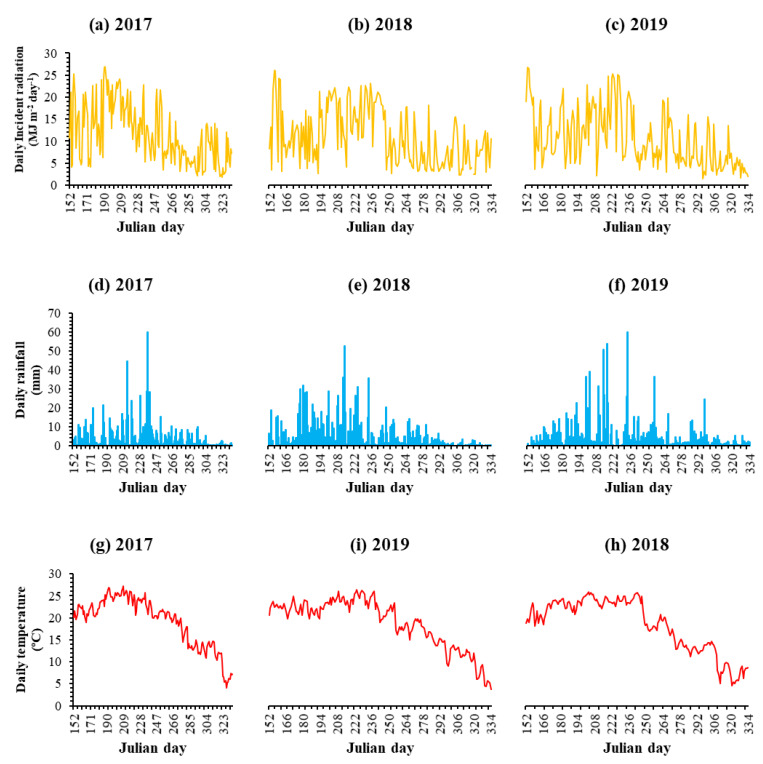
Daily incident radiation ((**a**) for 2017, (**b**) for 2018, and (**c**) for 2019), rainfall ((**d**) for 2017, (**e**) for 2018, and (**f**) for 2019), and temperature ((**g**) for 2017, (**h**) for 2018, and (**i**) for 2019) during the growth of summer soybean.

This study was carried out at the research site of Sichuan Agricultural University (29°98′ N, 103°59′ E), City Yaan, Province Sichuan, China. The study was performed for three consecutive years during the summer season of 2017, 2018, and 2019 with three replications for each treatment. The research area is categorized by a humid subtropical monsoon climate with a mean annual temperature of 16.2 °C. The average annual rainfall of this area is about 1200 mm, mostly occurring in the summer season (from June to August). Weather data (daily temperature and rainfall) of the research site during the cropping seasons are shown in Figure 6. The soil is characterized as fluvo-aquic soil [49], with a pH of 6.6. The contents of available nitrogen, phosphorus, potassium, and organic matter in the 0–20 cm soil layer were 0.32 g kg^−1^, 0.04 g kg^−1^, 0.38 g kg^−1^, and 29.8 g kg^−1^, respectively.

### 4.2. Experimental Materials and Design

**Figure 7:**
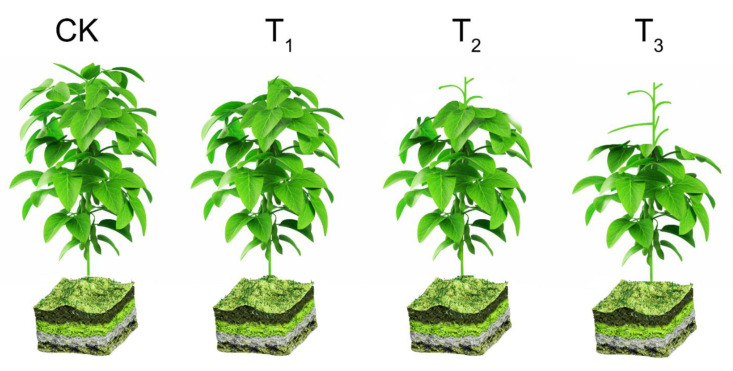
Pictorial representation of the soybean canopy as affected by different defoliation treatments under high-rainfall conditions during the growing season of 2017, 2018, and 2019. The CK refers to control treatment (no defoliation); T_1_, T_2_, and T_3_ refer to 15%, 30%, and 45%, defoliation, respectively, from the top of the soybean canopy.

The experimental design was a randomized complete block design (RCBD) with three replicates. After the harvesting of wheat, the soybean cultivar “Nandou-12 (determinate growth habit, lodging resistant, a variety of medium-maturity group; breeding material of Nanchong Academy of Agricultural Sciences in Sichuan Province)”, which is the famous cultivar of soybean in the southwest of China, was used as the experimental material [9,41]. In all years of the experiments, soybean was sown (at a seeding rate of 30 kg ha^−1^) in the second week of June at a planting population of 100,000 plants ha^−1^ using a plant-to-plant distance of 20 cm and a row-to-row distance of 50 cm and harvested in the third week of October. Four different treatments were organized in a randomized complete block design with three replications: no defoliation treatment was used as a control (CK), and three different defoliation treatments were applied at the pod initiation stage (R_3_) of soybean: T_1_ (15% defoliation); T_2_ (30% defoliation); and T_3_ (45% defoliation) from the top of the soybean canopy (Figure 7). Defoliation was performed (one time) manually using a leaf clipper (R_3_). These defoliation treatments were maintained by removing the different number of fully developed trifoliate (i.e., three trifoliates for T_1_, six trifoliates for T_2_, and nine trifoliates for T_3_) from the soybean plant. At R_3_, the total number of phytomeres in each soybean plant was 19 ± 3. We selected stage R_3_ for defoliation because the formation of reproductive parts (pods) starts from this stage in soybean [50]. In high-rainfall regions, soybean plants tend to lodge due to extra vegetative growth [51]. Therefore, we applied different defoliation treatments to evaluate the effect of reducing the leaf area on soybean, especially during the reproductive growth phase. All the growth stages were recorded by following the description of Fehr and Caviness (1977) (Table 6). The size of each experimental plot was 24 m^2^ (4 m × 6 m) and consisted of eight soybean rows spaced 0.50 m apart. Each experimental plot was separated by an uncropped space of two meters in width. At the time of soybean sowing, fertilizers were applied at 75 kg nitrogen ha^−1^ as urea, 40 kg phosphorus ha^−1^ as calcium superphosphate, and 10 kg potassium ha^−1^ as potassium sulfate. For seedbed preparations, conventional tillage, that is three cultivations with a tractor-mounted cultivator followed by planking, was practiced in the three years of the study. In this study, all farm machinery was owned by Sichuan Agricultural University, Chengdu, China. Weeds were controlled with hand hoeing, which was performed twice after the soybean sowing. Disease and pests were also well controlled using appropriate chemicals. Additionally, we used the central six rows of each treatment for plant sampling and measurements (with at least one meter away from the previous sampling); the first and last rows of each treatment were not selected due to border-row effects.

### 4.3. Measurements

#### 4.3.1. Leaf Area Index and Leaf Greenness

The leaf area index of soybean plants was determined at the pod initiation stage (R_3_), full pod stage (R_4_), seed initiation stage (R_5_), full seed stage (R_6_), and physiological maturity stage (R_7_). For this purpose, ten consecutive soybean plants were sampled from each experiment plot. The leaf area of every single leaf was measured by multiplying the leaf length and greatest leaf width with the crop-specific coefficient factor of 0.75 for soybean [52]. Then, the leaf area index of soybean plants was calculated using Equation (1) [53].

(1) $$\mathrm{Leaf}\;\mathrm{area}\;\mathrm{index}=\frac{\left(\mathrm{Leaf}\;\mathrm{area}\;\mathrm{per}\;\mathrm{plant}\;\times \mathrm{Plant}\;\mathrm{number}\;\mathrm{per}\;\mathrm{plot}\right)}{\mathrm{Plot}\;\mathrm{area}}$$

Moreover, the leaf greenness of soybean leaves at all sampling stages (R_3_, R_4_, R_5_, R_6_, and R_7_) was measured using SPAD-502 (Soil Plant Analysis Development, Konica Minolta, Japan). For this purpose, three fully developed trifoliates from the middle of the soybean canopy (in total, nine individual leaves) were selected to measure the leaf greenness of soybean plants, and the average was calculated. In all treatments, we did not use the young leaves for the measurement of leaf greenness; and we measured the leaf greenness from the same phytomer rank (8th phytomer rank) of all sampled plants in all treatments.

#### 4.3.2. Photosynthetically Active Radiation Transmittance and Photosynthetic Parameters

The photosynthetically active radiation (PAR) was determined at R_5_ because soybean achieved the maximum leaf area at R_5_ [43], using the quantum sensors (LI-191SA, LICOR Inc., Lincoln, NE, USA) equipped with a digital data logger. To measure the PAR, first, sensors were placed at the top of the soybean canopy and then at the ground level. The PAR of each treatment was determined three times, from 10:30 to 11:30 h on a sunny day. Then, the PAR transmittance was estimated using Equation (2) [9]:

(2) $$\mathrm{PAR}\;\mathrm{transmittance}\;\left(\%\right)=\frac{{\mathrm{PAR}}_{G}}{{\mathrm{PAR}}_{T}}\times 100$$

where PAR*_T_* is the PAR above the soybean canopy and PAR*_G_* is the PAR at ground level.

Furthermore, the photosynthetic parameters of soybean leaves were measured at R_5_ using Li-6400 (LI-COR Inc., Lincoln, NE, USA). For this purpose, three fully developed individual leaves from the middle of the soybean canopy were selected to measure the photosynthetic parameters. All photosynthetic measurements were taken from 11:30 to 13:00 h under a steady light intensity of 1000 μmol m^−2^ s^−1^, a temperature of 25 °C, and a carbon dioxide concentration of 400 µmol mol^−1^. Note, we only selected those soybean leaves that were receiving the same levels of solar radiation in all treatments.

#### 4.3.3. Dry Matter and Crop Growth Rate

At R_3_, R_4_, R_5_, R_6_, and R_7_, after the measurement of the leaf area index, we used the same plant samples for dry matter analysis. First, we divided the plant samples into vegetative (stem + leaves, g m^−2^) and reproductive parts (pods + seeds, g m^−2^). After that, all samples were placed in an oven at 65 °C to obtain the constant weight of all parts and then weighed. Additionally, at all sampling stages, the total dry matter accumulation (TDM, kg ha^−1^) was estimated from the summation of the dry matter of vegetative parts and reproductive parts [16].

The crop growth rate (g m^−2^ day^−1^) of soybean was calculated among R_3_–R_4_, R_4_–R_5_, R_5_–R_6_, and R_6_–R_7_. The crop growth rate and reproductive growth rate of soybean were measured using Equation (3) [54].

(3) $$\mathrm{Crop}\;\mathrm{growth}\;\mathrm{rate}=\frac{{\mathrm{TDM}}_{2}-{\mathrm{TDM}}_{1}}{T_{2}-T_{1}}$$

where TDM_1_ and TDM_2_ are the total dry matter (vegetative dry matter + reproductive dry matter) of soybean plants at Stages 1 and 2, respectively. T_1_ is the time of the first sampling, and T_2_ is the time of the second sampling.

#### 4.3.4. Yield and Yield Components

At soybean maturity, the four-meter square area was harvested by cutting soybean plants at ground level from all treatments and sun dried for the next seven days. After sun drying, all pods were separated from the soybean plants, and the number of pods m^−2^ was counted, as well as the average determined. Then, all pods were manually threshed, and the average number of seeds m^−2^ was determined, then all the seeds of the sampled plants were weighed to calculate the seed yield of each plant and converted into kg ha^−1^. Five lots of one hundred seeds from the bulk seed lot of each treatment were oven dried at 65 °C till constant weight, and then, the seed weight (SW) (in mg) was recorded using an electrical balance, then the average was calculated.

### 4.4. Statistical Analysis

Statistical analyses were conducted using Statistix 8.1. Significant differences were measured by using ANOVA in combination with the LSD (least significance difference) test. The significance of the differences was evaluated at the *p* < 0.05 level. Tables report the means and the standard errors of the calculated means based on the three replicates of each treatment. The Pearson correlation was used to analyze the relationship of the growth and yield components with the leaf area index at R_5_; we selected the R_5_ stage because researchers have reported that soybean achieves the maximum leaf area index at this stage [43,50].

## Figures and Tables

**Table 1:** Leaf greenness of soybean at different phenological stages as affected by different defoliation treatments during the summer season of 2017, 2018, and 2019.

Year	Treatment	Growth Stages				
		R_3_	R_4_	R_5_	R_6_	R_7_
2017	CK	25.5 ± 0.5 ^NS^	35.4 ± 1.0 ^a^	42.2 ± 1.8 ^a^	31.9 ± 1.8 ^b^	29.5 ± 2.4 ^ab^
	T_1_	25.1 ± 0.6	32.1 ± 1.6 ^ab^	38.1 ± 3.0 ^ab^	36.5 ± 2.8 ^a^	35.9 ± 3.4 ^a^
	T_2_	24.6 ± 1.4	30.2 ± 1.5 ^ab^	33.7 ± 0.8 ^bc^	28.8 ± 1.5 ^c^	24.4 ± 1.2 ^b^
	T_3_	25.1 ± 0.7	26.6 ± 1.6 ^b^	28.5 ± 1.2 ^c^	23.1 ± 1.2 ^d^	20.6 ± 1.9 ^b^
2018	CK	27.7 ± 1.7 ^NS^	38.6 ± 2.5 ^a^	43.8 ± 2.2 ^a^	39.6 ± 2.7 ^a^	32.6 ± 0.2 ^b^
	T_1_	27.3 ± 0.6	36.2 ± 1.1 ^ab^	42.0 ± 2.8 ^ab^	41.1 ± 3.1 ^ab^	39.9 ± 2.7 ^a^
	T_2_	26.5 ± 0.5	30.9 ± 2.2 ^bc^	34.6 ± 1.1 ^bc^	36.9 ± 2.6 ^ab^	30.8 ± 1.3 ^b^
	T_3_	26.1 ± 1.3	28.5 ± 1.2 ^c^	32.1 ± 1.5 ^c^	33.5 ± 1.4 ^b^	28.6 ± 1.1 ^b^
2019	CK	25.0 ± 1.1 ^NS^	33.2 ± 1.6 ^a^	36.8 ± 1.7 ^a^	28.5 ± 1.4 ^ab^	25.9 ± 1.7 ^b^
	T_1_	24.4 ± 0.3	31.9 ± 1.2 ^ab^	34.2 ± 2.3 ^ab^	32.9 ± 3.6 ^a^	31.2 ± 2.5 ^a^
	T_2_	26.0 ± 1.2	29.1 ± 1.6 ^bc^	31.4 ± 1.5 ^ab^	25.4 ± 1.1 ^ab^	21.5 ± 1.1 ^bc^
	T_3_	25.2 ± 0.7	27.4 ± 1.1 ^c^	28.1 ± 1.6 ^b^	23.3 ± 1.5 ^b^	19.1 ± 1.5 ^c^

Treatment codes represent 100% leaf area (CK: control), 85% leaf area (T_1_), 70% leaf area (T_2_), and 55% leaf area (T_3_) from the soybean canopy. Means are averages over three replicates ± the standard error of the mean. Means that do not share the same letters in a column differ significantly at *p* < 0.05 using least significant differences (LSDs), calculated separately for each year; NS = nonsignificant.

**Table 2:** Light transmittance, photosynthetic rate (*Pn*), transpiration rate (*Tr*), stomatal conductance (*Gs*), and intercellular CO_2_ concentration (*Ci*) of soybean at the seed initiation stage (R_5_) as affected by different defoliation treatments during the summer season of 2017, 2018, and 2019.

Year	Treatment	Light Transmittance	*Pn*	*Tr*	*Gs*	*Ci*
		(%)	(μmol CO_2_ m^−2^ s^−1^)	(mmol H_2_O m^−2^ s^−1^)	(mol H_2_O m^−2^ s^−1^)	(μmol CO_2_ m^−2^ s^−1^)
2017	CK	6.7 ± 0.6 ^a^	11.8 ± 0.6 ^c^	3.0 ± 0.3 ^c^	0.5 ± 0.0 ^c^	321.3 ± 7.0 ^a^
	T_1_	9.4 ± 0.7 ^a^	13.6 ± 0.8 ^b^	3.7 ± 0.5 ^bc^	0.6 ± 0.0 ^b^	279.4 ± 8.6 ^b^
	T_2_	13.3 ± 1.2 ^b^	15.5 ± 0.4 ^a^	4.4 ± 0.3 ^ab^	0.7 ± 0.0 ^b^	257.9 ± 16.2 ^bc^
	T_3_	15.7 ± 1.0 ^b^	17.1 ± 0.2 ^a^	5.2 ± 0.3 ^a^	0.8 ± 0.0 ^a^	241.2 ± 9.7 ^c^
2018	CK	10.6 ± 0.8 ^d^	12.3 ± 0.9 ^c^	2.7 ± 0.3 ^c^	0.5 ± 0.0 ^c^	354.7 ± 23.7 ^a^
	T_1_	13.8 ± 1.2 ^c^	15.7 ± 0.6 ^b^	3.6 ± 0.5 ^bc^	0.7 ± 0.0 ^b^	321.0 ± 7.5 ^ab^
	T_2_	19.2 ± 0.9 ^b^	17.4 ± 0.4 ^b^	4.3 ± 0.3 ^ab^	0.6 ± 0.0 ^b^	295.8 ± 13.5 ^b^
	T_3_	23.3 ± 2.4 ^a^	19.9 ± 0.5 ^a^	5.0 ± 0.3 ^a^	0.8 ± 0.0 ^a^	273.6 ± 11.2 ^b^
2019	CK	7.3 ± 0.6 ^d^	10.3 ± 0.1 ^d^	2.7 ± 0.4 ^d^	0.3 ± 0.0 ^c^	328.1 ± 24.9 ^a^
	T_1_	11.7 ± 1.0 ^c^	15.8 ± 0.2 ^c^	3.7 ± 0.2 ^c^	0.4 ± 0.0 ^c^	280.1 ± 13.0 ^ab^
	T_2_	16.3 ± 0.8 ^b^	15.5 ± 0.2 ^b^	4.5 ± 0.3 ^b^	0.5 ± 0.0 ^b^	247.0 ± 6.9 ^b^
	T_3_	21.0 ± 1.0 ^a^	18.7 ± 0.0 ^a^	5.5 ± 0.3 ^a^	0.7 ± 0.0 ^a^	234.6 ± 22.5 ^b^

Treatment codes represent 100% leaf area (CK: control), 85% leaf area (T_1_), 70% leaf area (T_2_), and 55% leaf area (T_3_) from the soybean canopy. Means are averages over three replicates ± the standard error of the mean. Means that do not share the same letters in a column differ significantly at *p* < 0.05 using least significant differences (LSDs), calculated separately for each year.

**Table 3:** Dry matter accumulation in vegetative (leaves + stems; g m^−2^) and reproductive (pods + seeds; g m^−2^) organs of soybean at different phenological stages as affected by different defoliation treatments during the summer season of 2017, 2018, and 2019.

Year	Treatment	Growth Stages									
		R_3_		R_4_		R_5_		R_6_		R_7_	
		Vegetative Parts	Reproductive Parts	Vegetative Parts	Reproductive Parts	Vegetative Parts	Reproductive Parts	Vegetative Parts	Reproductive Parts	Vegetative Parts	Reproductive Parts
2017	CK	271.2 ± 10.2 ^a^	32.3 ± 2.8 ^NS^	323.2 ± 9.0 ^a^	53.2 ± 1.0 ^b^	401.8 ± 4.8 ^a^	116.7 ± 7.9 ^b^	386.2 ± 5.0 ^a^	223.1 ± 7.0 ^b^	288.1 ± 7.8 ^a^	365.5 ± 2.6 ^b^
	T_1_	234.3 ± 17.6 ^b^	31.8 ± 2.7	289.3 ± 7.3 ^b^	71.7 ± 5.2 ^a^	379.5 ± 9.7 ^b^	149.5 ± 4.9 ^a^	379.1 ± 18.3 ^a^	257.1 ± 8.5 ^a^	301.6 ± 10.7 ^a^	401.8 ± 3.2 ^a^
	T_2_	185.6 ± 5.1 ^c^	28.2 ± 1.2	233.4 ± 4.7 ^c^	41.6 ± 1.9 ^c^	302.4 ± 9.2 ^c^	85.1 ± 2.0 ^c^	301.0 ± 6.4 ^b^	162.0 ± 1.7 ^c^	234.5 ± 8.2 ^b^	259.8 ± 3.0 ^c^
	T_3_	143.6 ± 12.4 ^d^	27.6 ± 2.7	179.9 ± 9.9 ^d^	34.7 ± 1.6 ^c^	236.0 ± 10.0 ^d^	64.9 ± 5.6 ^d^	252.6 ± 7.0 ^c^	111.2 ± 3.0 ^d^	185.8 ± 13.2 ^c^	198.8 ± 5.6 ^d^
2018	CK	283.9 ± 8.9 ^a^	41.2 ± 1.5 ^NS^	347.9 ± 3.0 ^a^	80.6 ± 1.6 ^b^	431.8 ± 8.3 ^a^	133.5 ± 1.8 ^b^	415.8 ± 5.3 ^a^	245.4 ± 4.7 ^b^	327.8 ± 3.5 ^a^	419.7 ± 2.5 ^b^
	T_1_	251.5 ± 5.7 ^b^	42.2 ± 1.2	318.7 ± 1.9 ^b^	91.8 ± 1.1 ^a^	396.7 ± 6.0 ^b^	165.0 ± 4.9 ^a^	401.2 ± 3.3 ^a^	282.0 ± 4.5 ^a^	334.1 ± 2.5 ^a^	455.8 ± 2.5 ^a^
	T_2_	189.4 ± 7.7 ^c^	41.1 ± 1.3	250.4 ± 5.6 ^c^	66.0 ± 3.6 ^c^	322.7 ± 8.0 ^c^	103.8 ± 3.4 ^c^	317.4 ± 2.8 ^b^	191.1 ± 1.4 ^c^	269.1 ± 4.2 ^b^	308.6 ± 2.0 ^c^
	T_3_	146.5 ± 1.6 ^d^	40.8 ± 0.7	207.7 ± 4.7 ^d^	50.8 ± 1.7 ^d^	274.9 ± 9.4 ^d^	69.8 ± 2.9 ^d^	266.0 ± 3.6 ^c^	140.2 ± 4.4 ^d^	221.1 ± 9.5 ^c^	241.4 ± 10.2 ^d^
2019	CK	296.9 ± 7.2 ^a^	27.6 ± 2.4 ^NS^	350.1 ± 6.1 ^a^	51.9 ± 1.5 ^b^	463.3 ± 8.6 ^a^	92.8 ± 4.9 ^b^	414.2 ± 2.2 ^a^	236.9 ± 5.8 ^b^	299.7 ± 3.7 ^b^	426.3 ± 7.2 ^b^
	T_1_	254.7 ± 6.4 ^b^	26.7 ± 3.7	303.6 ± 4.5 ^b^	68.5 ± 4.4 ^a^	412.4 ± 8.2 ^b^	135.0 ± 3.5 ^a^	395.4 ± 5.1 ^b^	269.0 ± 4.6 ^a^	317.7 ± 3.0 ^a^	446.9 ± 1.9 ^a^
	T_2_	214.7 ± 6.1 ^c^	26.1 ± 2.3	259.3 ± 5.2 ^c^	44.8 ± 2.1 ^bc^	340.0 ± 3.9 ^c^	94.9 ± 3.0 ^b^	354.3 ± 5.1 ^c^	151.3 ± 8.0 ^c^	245.3 ± 2.6 ^c^	313.1 ± 8.1 ^c^
	T_3_	172.6 ± 5.9 ^d^	26.6 ± 3.3	209.9 ± 7.1 ^d^	34.6 ± 2.8 ^c^	295.6 ± 2.4 ^d^	53.8 ± 3.7 ^c^	274.7 ± 4.1 ^d^	124.8 ± 3.7 ^d^	198.9 ± 6.7 ^d^	229.7 ± 3.5

Treatment codes represent 100% leaf area (CK: control), 85% leaf area (T_1_), 70% leaf area (T_2_), and 55% leaf area (T_3_) from the soybean canopy. Means are averages over three replicates ± the standard error of the mean. Means that do not share the same letters in a column differ significantly at *p* < 0.05 using least significant differences (LSDs), calculated separately for each year; NS = nonsignificant.

**Table 4:** The crop growth rate (g m^−2^ day^−1^) of soybean plants at different phenological stages as affected by different defoliation treatments during the summer season of 2017, 2018, and 2019.

Year	Treatment	Growth Stages			
		R_3_–R_4_	R_4_–R_5_	R_5_–R_6_	R_6_–R_7_
2017	CK	9.1 ± 0.6 ^ab^	15.8 ± 0.8 ^a^	13.0 ± 0.6 ^b^	6.3 ± 0.7 ^b^
	T_1_	11.9 ± 1.2 ^a^	18.7 ± 1.0 ^a^	15.3 ± 1.0 ^a^	9.6 ± 1.3 ^a^
	T_2_	7.7 ± 0.9 ^bc^	12.5 ± 1.0 ^b^	10.8 ± 0.7 ^bc^	4.5 ± 0.8 ^bc^
	T_3_	5.4 ± 0.5 ^c^	9.6 ± 0.8 ^b^	9.0 ± 0.7 ^bc^	3.0 ± 0.7 ^c^
2018	CK	11.5 ± 0.4 ^a^	15.2 ± 1.1 ^ab^	12.0 ± 1.3 ^ab^	10.8 ± 0.7 ^ab^
	T_1_	13.0 ± 0.7 ^a^	16.8 ± 1.1 ^a^	15.2 ± 0.9 ^a^	13.3 ± 0.8 ^a^
	T_2_	9.5 ± 0.4 ^b^	12.2 ± 0.6 ^bc^	10.3 ± 1.5 ^bc^	8.7 ± 0.7 ^bc^
	T_3_	7.9 ± 0.5 ^b^	9.6 ± 1.0 ^c^	7.7 ± 0.9 ^c^	7.0 ± 0.5 ^c^
2019	CK	11.1 ± 0.5 ^a^	15.4 ± 0.9 ^ab^	11.9 ± 0.9 ^b^	9.4 ± 0.9 ^ab^
	T_1_	12.9 ± 0.8 ^a^	17.5 ± 0.8 ^a^	14.6 ± 0.4 ^a^	12.5 ± 1.3 ^a^
	T_2_	9.0 ± 0.6 ^b^	13.1 ± 0.4 ^b^	8.8 ± 0.6 ^c^	6.6 ± 1.0 ^bc^
	T_3_	6.5 ± 0.3 ^c^	10.5 ± 0.6 ^c^	6.3 ± 0.6 ^d^	3.6 ± 0.7 ^c^

Treatment codes represent 100% leaf area (CK: control), 85% leaf area (T_1_), 70% leaf area (T_2_), and 55% leaf area (T_3_) from soybean canopy. Means are averages over three replicates ± the standard error of the mean. Means that do not share the same letters in a column differ significantly at *p* < 0.05 using least significant differences (LSDs), calculated separately for each year; NS = nonsignificant.

**Table 5:** Yield and yield components of soybean as affected by different defoliation treatments during the summer season of 2017, 2018, and 2019.

Year	Treatment	Yield and Yield Components			
		Number of Pods	Number of Seeds	100-Seed Weight	Seed Yield
		(m^−2^)	(m^−2^)	(g)	(kg ha^−1^)
2017	CK	803.1 ± 36.4 ^b^	1139.6 ± 64.7 ^b^	18.1 ± 0.2 ^NS^	2069.0 ± 130.3 ^b^
	T_1_	1022.5 ± 47.8 ^a^	1247.6 ± 52.7 ^a^	18.0 ± 0.1	2243.9 ± 100.2 ^a^
	T_2_	671.8 ± 53.6 ^bc^	956.3 ± 49.9 ^c^	18.2 ± 0.3	1743.6 ± 95.4 ^c^
	T_3_	524.7 ± 32.3 ^c^	759.6 ± 57.0 ^d^	18.4 ± 0.2	1396.7 ± 97.6 ^d^
2018	CK	912.0 ± 37.3 ^b^	1291.8 ± 36.1 ^b^	17.6 ± 0.3 ^NS^	2273.2 ± 98.3 ^b^
	T_1_	1072.7 ± 46.6 ^a^	1424.3 ± 61.5 ^a^	17.3 ± 0.3	2460.5 ± 118.5 ^a^
	T_2_	715.8 ± 69.3 ^c^	1062.8 ± 42.2 ^c^	17.8 ± 0.4	1897.3 ± 103.9 ^c^
	T_3_	563.7 ± 44.7 ^d^	895.6 ± 47.2 ^d^	17.7 ± 0.1	1584.8 ± 72.6 ^d^
2019	CK	756.4 ± 24.3 ^b^	1112.7 ± 4.1 ^b^	18.1 ± 0.2 ^NS^	2018.5 ± 55.7 ^b^
	T_1_	942.9 ± 39.7 ^a^	1263.3 ± 3.6 ^a^	17.8 ± 0.3	2250.4 ± 72.9 ^a^
	T_2_	655.1 ± 24.7 ^b^	840.7 ± 4.5 ^c^	18.2 ± 0.2	1534.7 ± 93.4 ^c^
	T_3_	494.4 ± 30.1 ^c^	713.8 ± 3.5 ^d^	18.3 ± 0.2	1302.7 ± 64.2 ^d^

Treatment codes represent 100% leaf area (CK: control), 85% leaf area (T_1_), 70% leaf area (T_2_), and 55% leaf area (T_3_) from soybean canopy. Means are averages over three replicates ± the standard error of the mean. Means that do not share the same letters in a column differ significantly at *p* ≤ 0.05 using least significant differences (LSDs), calculated separately for each year; NS = nonsignificant.

**Table 6:** Soybean phenological stages and growth phases as recorded during the summer season of 2017, 2018, and 2019 at the research site of Sichuan Agricultural University, Yaan, China.

Phenological Stage	Growth Phase	Julian Day		
		2017	2018	2019
Seed emergence (VE)	Germination	165	167	172
Fifth-trifoliate (V_5_)	Vegetative	194	194	199
Flower-initiation (R_1_)	Pre-reproductive	216	216	224
Pod-initiation (R_3_)	Reproductive	228	234	240
Full pod (R_4_)	Reproductive	236	243	247
Seed-initiation (R_5_)	Reproductive	245	252	257
Full seed (R_6_)	Reproductive	252	260	265
Physiological maturity (R_7_)	Reproductive	259	268	273
Full maturity (R_8_)	Reproductive	282	289	296

## Data Availability

The data will be provided by the corresponding author upon demand.
